# Research on a Minor Organism can also be Benefit the World: The Fascinating Cellular Slime Mold *Dictyostelium discoideum*

**DOI:** 10.14789/jmj.JMJ24-0021-R

**Published:** 2024-09-11

**Authors:** YUZURU KUBOHARA

**Affiliations:** 1 Laboratory of Health and Life Science, Juntendo University Graduate School of Health and Sports Science, Chiba, Japan; 1 Laboratory of Health and Life Science, Juntendo University Graduate School of Health and Sports Science, Chiba, Japan

**Keywords:** cellular slime mold, *Dictyostelium discoideum*, DIF-1, drug development

## Abstract

In 1985, when I entered the Graduate School of Science at Kyoto University, I began my research on cellular slime molds, a group of soil microorganisms. The cellular slime mold *Dictyostelium discoideum* is studied globally as a model organism for cell and developmental biology. I was conducting basic biological research into cell differentiation and migration using *D. discoideum*, and during this process, our research group made a discovery with potential implications for drug development. Specifically, we found that a chlorinated polyketide named differentiation-inducing factor 1 (DIF-1), derived from *D. discoideum*, exhibits antitumor activity. Based on this discovery, I began elucidating the mechanism of the antitumor action of DIF-1 and developing anticancer drugs using DIF-1 as a lead compound. During this period, in 1991, I obtained my Ph.D. in research related to *D. discoideum* cell differentiation, and subsequently served as a Japan Society for the Promotion of Science (JSPS) Special Research Fellow before joining the Institute for Molecular and Cellular Regulation (IMCR) at Gunma University in 1993. I then joined the Graduate School of Health and Sports Sciences at Juntendo University in 2015, where I have been until 2024. Throughout this period, I continued my research on DIF-1 and discovered that DIF-1 and its derivatives possess various biological activities ─ such as anti-diabetic, immunoregulatory, anti-bacterial, and anti-malarial activities ─ that could be applicable in drug development. In this review, I aim to present a segment of both our fundamental and applied research on *D. discoideum* and DIF-1.

## Introduction

After graduating from Osaka University (Department of Biology, Faculty of Science) and working for a while at a private company, I enrolled in the Graduate School of Science at Kyoto University in 1985 and started my research (on cellular slime molds) at the Cell Biology Laboratory.

**Figure 1:**
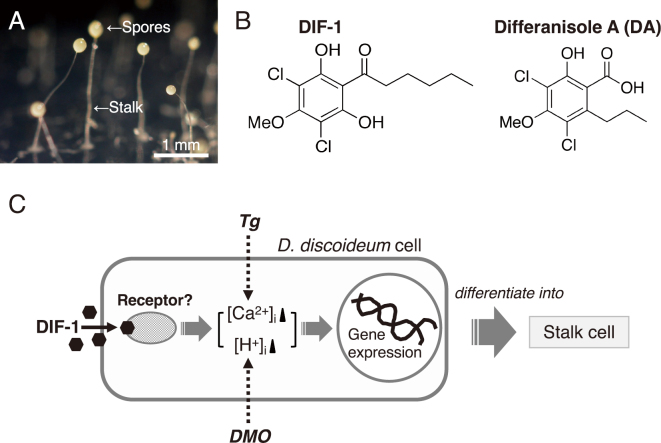
(A) Fruiting bodies of the cellular slime mold *Dictyostelium discoideum*, each consisting of spores and a multicellular stalk. (B) Chemical structures of DIF-1 and differanisole A (DA). DIF-1, originally discovered in *D. discoideum*, acts as a stalk cell differentiation-inducing factor^9)^, while DA is an antitumor agent found in a fungal strain of the *Chaetomium* genus^14)^. (C) Schematic diagram illustrating how DIF-1 induces stalk cell differentiation (revised figure from ref. 12). DIF-1 may induce stalk cell differentiation by increasing [Ca^2+^]_i_ and [H^+^]_i_ possibly through a putative DIF-receptor in *D. discoideum* cells. Co-administration of Tg and DMO can replicate the effects of DIF-1 and induce stalk cell differentiation in the absence of DIF-1.

Cellular slime molds are eukaryotic microorganisms that live, for example, under fallen leaves in forests around the world. They form fruiting bodies resembling those of fungi. However, cellular slime molds and fungi belong to different taxonomic kingdoms^1-3)^. The life cycle of cellular slime molds is quite simple. For instance, vegetative amoebae of the cellular slime mold *Dictyostelium discoideum* germinate from spores and multiply by feeding on bacteria. Upon starvation, the amoebae aggregate to form a multicellular body, which eventually develops into a fruiting body consisting of spores and a multicellular stalk (Figure 1A)^4)^. Due to its simple life cycle and ease of handling, *D. discoideum* has served as a model organism for studying cell and developmental biology, including cell growth, cell differentiation, chemotactic cell movement, morphogenesis, and autophagy^4-7)^. However, the number of researchers studying slime molds worldwide is relatively small compared to those working on other model organisms, and cellular slime molds may be considered a less prominent model organism globally. Nevertheless, in recent years, cellular slime molds have been garnering attention as an untapped resource for drug discovery, and indeed, many new compounds derived from cellular slime molds have been discovered^8)^. In particular, the history of the discovery of the diverse biological activities of DIF- 1 (differentiation-inducing factor 1) (Figure 1B), originally found in *D. discoideum*, along with DIF- 1’s derivatives, deserves special mention.

## DIF-1, a stalk cell differentiation- inducing factor in *D. discoideum*

In 1987, while I was researching the mechanism of stalk cell differentiation in *D. discoideum* at Kyoto University Graduate School of Science, a British research group identified a chlorinated polyketide compound, DIF-1 (Figure 1B), which induces stalk cell differentiation in *D. discoideum*^9-11)^. However, the mechanism underlying the action of DIF-1 remained unclear for some time. We later discovered that thapsigargin (Tg) and 5,5-dimethyl- 2,4-oxazolidinedione (DMO) can induce stalk cell differentiation in the absence of DIF-1 (i.e., in a DIF-deficient strain) (Figure 1C)^12)^. Tg inhibits the Ca^2+^-ATPase present in the endoplasmic and sarcoplasmic reticula, thereby raising the intracellular calcium concentration ([Ca^2+^]_i_), while DMO increases the intracellular proton concentration ([H^+^]_i_). Therefore, our results suggested that DIF-1 induces stalk cell differentiation by potentially increasing [Ca^2+^]_i_ and [H^+^]_i_ through a putative DIF receptor (Figure 1C)^12)^. Incidentally, even after around 35 years since the discovery of DIF-1, the receptor for DIF-1 has not been identified^13)^.

## Differanisole A (DA)-an antitumor agent found in a fungus-and the discovery of the antitumor activity of DIF-1

**Figure 2:**
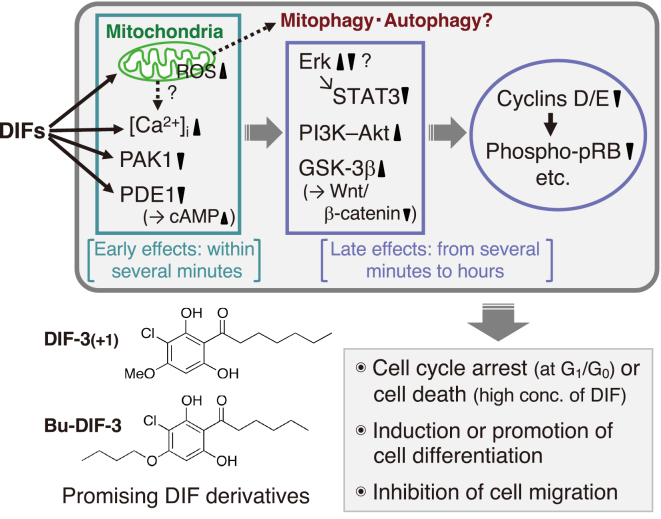
Proposed scheme for the antitumor effects of DIFs (revised figure from ref. 8). DIFs at concentrations ranging from several micromolars to several tens of micromolars trigger the indicated intracellular phenomena, leading to cell cycle arrest at G1/G0, induction/promotion of cell differentiation, and inhibition of cell migration^8, 16-27)^. Notably, at appropriate concentrations, DIFs have inhibited cell growth in all tested tumor cell lines both in vitro and in vivo, and at higher concentrations, they have induced caspase-independent cell death in vitro^18, 21, 25)^. DIFs also induce re-differentiation (erythroid differentiation) into hemoglobin-producing cells in murine and human leukemia (B8 and K562) cells^16, 20)^ and promote retinoic acid-induced granulocyte differentiation in human leukemia HL-60 cells in vitro^19)^. Additionally, DIFs suppress the migration and metastasis of certain cancer cells both in vitro and in vivo^24, 26, 27)^. Chemical structure-activity relationship (SAR) analyses have revealed that several DIFs, such as DIF-3(+1) and Bu-DIF-3, show promise as lead compounds for the development of anticancer drugs^8, 17)^. Abbreviations: ROS, reactive oxygen species; PAK1, p21-activated kinase 1; PDE1, calmodulin-dependent cAMP/cGMP phosphodiesterase; Erk, extracellular signal-regulated kinase; STAT3, signal transducer and activator of transcription 3; PI3K, phosphatidylinositol 3-kinase; GSK-3β, glycogen synthase kinase-3β; pRB, retinoblastoma protein.

Two years before the discovery of DIF-1^9)^, while I was conducting DIF research at Kyoto University Graduate School of Science, a Japanese research group at RIKEN (Institute of Physical and Chemical Research) screened for anticancer drug candidates derived from various fungi. They isolated a compound called differanisole A (DA) from a species of the *Chaetomium* genus (Figure 1B); DA was found to suppress the proliferation of murine erythroleukemia B8 cells and induce re-differentiation of B8 cells into hemoglobin-producing cells in vitro^14)^. At the time of DA’s discovery, we were unaware of its existence because it was discovered in a different field of research. However, in the 1990s, both RIKEN and our research group focused on the structures of DIF-1 and DA. We discovered that DA can induce stalk cell differentiation in *D. discoideum* in vitro^15)^ and that DIF-1 can suppress the proliferation and induce erythroid differentiation of B8 cells and human K562 leukemia cells in vitro^16)^. Because DIF-1 exhibited stronger antitumor activities than DA, we began analyzing the mechanisms underlying the antitumor effects of DIF-1 and its derivatives (DIFs) and developing anticancer drugs using DIFs as lead compounds^8)^. Currently, several research groups, other than ours, are analyzing the mechanisms of the antitumor effects of DIFs. Figure 2 illustrates the schematic diagram of the antitumor effects of DIFs elucidated so far, along with the chemical structures of two promising DIF derivatives^8, 17)^. In summary, DIFs are thought to induce cell cycle arrest or cell death, induce or promote cell differentiation, and inhibit cell migration (invasion/metastasis) through the indicated intracellular mechanisms^18-27)^.

Note that, amidst these studies, I obtained a Ph.D. degree in research related to *D. discoideum* cell differentiation in 1991. Then, after serving as a JSPS Special Research Fellow for 2 years, I joined Gunma University’s IMCR in 1993. Additionally, during my time at IMCR, I pursued studies abroad at the Medical Research Council (MRC) Laboratory of Molecular Biology in Cambridge, UK from 1999 to 2000 as a Ministry of Education Overseas Research Fellow (Zaigai-kenkyuin).

## Glucose uptake-promoting activity of DIF-1

**Figure 3:**
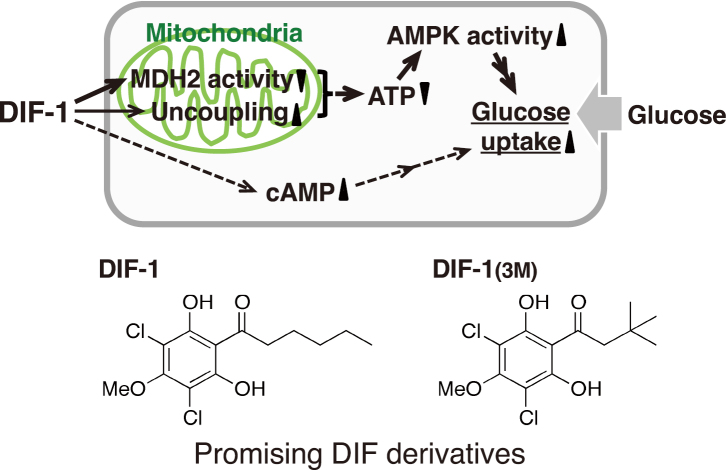
Proposed scheme for the glucose uptake-promoting effect of DIF-1 (revised figure from ref. 33). Our findings have demonstrated that DIF-1 at concentrations of 10-20 μM promotes glucose uptake, at least in part, by uncoupling mitochondrial activity and inhibiting mitochondrial malate dehydrogenase (MDH2) activity, thereby reducing ATP production and activating AMP kinase (AMPK) in mammalian cells^8, 28, 31, 33)^. It has also been suggested that DIF-1 promotes glucose uptake partly through an increase in cellular cAMP levels^32)^. SAR analyses have revealed that both DIF-1 and DIF-1(3M) show promise as lead compounds for the development of anti-diabetes and anti-obesity drugs^8, 28, 30)^.

In 2007, while investigating the toxicity of DIF-1 against normal mammalian cells to develop anticancer drugs using DIF-1 as a lead compound, I serendipitously discovered that DIF-1 promotes glucose uptake and metabolism in vitro in mammalian cells^28)^. Furthermore, we demonstrated that oral administration of DIF-1 reduces blood glucose levels in streptozotocin-treated diabetic rats^29)^. These findings suggest that DIF-1 and its derivatives may have therapeutic potential for treating obesity and/or diabetes. Figure 3 illustrates the schematic diagram of the glucose uptake-promoting effects of DIF-1 discovered thus far, along with the chemical structures of two promising DIF derivatives^28-33)^. It is noteworthy that DIF-1 and DIF-1(3M) exhibit weaker antitumor activities compared to other DIFs such as DIF-3(+1) and Bu-DIF-3 (Figure 2)^8, 28, 30)^. Therefore, DIF-1 and DIF-1(3M) are expected to serve as lead compounds for the development of anti-obesity and/or anti- diabetes drugs with few or no side effects^8)^.

## Immunoregulatory activities of DIFs

As we have come to understand that DIFs are multifunctional molecules, we have decided to systematically assess the biological activity of DIFs alongside the development of DIF-derived anticancer and anti-obesity/anti-diabetes drugs.

**Figure 4:**
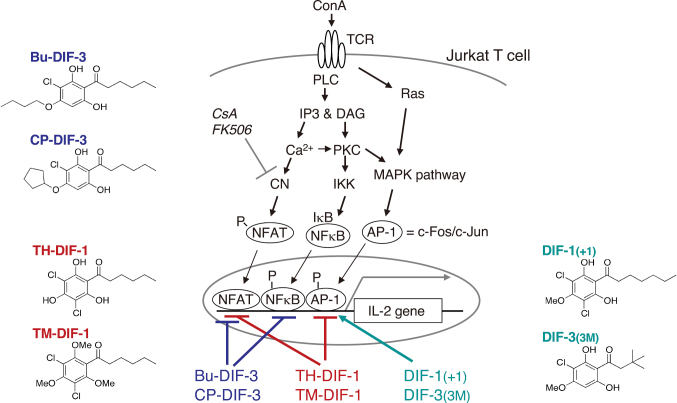
Proposed scheme for the immune-regulatory effects of DIFs on ConA-induced IL-2 production in Jurkat T cells (revised figure from ref. 37). The mitogen ConA stimulates IL-2 production via TCR and subsequent activation (phosphorylation or dephosphorylation) of the MAPK/AP-1, IKK/NFκB, and CN/NFAT pathways in T-lymphocytes, whereas the immunosuppressive drugs cyclosporin A (CsA) and FK506 inhibit CN via the immunophilins^52-55)^. DIFs at several micromolar levels affect ConA-induced IL-2 production in vitro in Jurkat T cells. For example, Bu-DIF-3 and CP-DIF-3 suppress IL-2 expression by inhibiting NFAT and NFκB activities, whereas TH-DIF-1 and TM-DIF-1 suppress it, at least in part, by inhibiting NFAT and AP-1 activities^36)^. In contrast, DIF-1(+1) and DIF-3(3M) promote ConA-induced IL-2 mRNA expression, at least in part, by enhancing AP-1 activity^37)^. Abbreviations: ConA, concanavalin A; TCR, T-cell receptor; PLC, phospholipase C; IP3, inositol 1,4,5-trisphosphate; CN, calcineurin; DAG, diacylglycerol; PKC, protein kinase C; IKK, IκB kinase; MAPK, mitogen-activated protein kinase; NFAT, nuclear factor of activated T-cell; NFκB, nuclear factor kappa B; AP-1, activator protein-1, a transcription factor formed from a heterodimer of c-Fos and c-Jun. P- denotes a phosphate group.

The mitogen concanavalin A (ConA) can stimulate IL-2 production in vitro in Jurkat T cells, a model cell line for human T-lymphocytes^34, 35)^. We examined the effects of DIFs on ConA-induced IL-2 production (CIIP) in Jurkat T cells and found that DIFs such as Bu-DIF-3 and CP-DIF-3 suppress CIIP, at least in part, by inhibiting NFAT and NF*κ*B activities, while DIFs such as TH-DIF-1 and TM-DIF-1 suppress CIIP, at least in part, by inhibiting NFAT and AP-1 activities^36)^. Conversely, DIFs such as DIF-1(+1) and DIF-3(3M) promote CIIP, at least in part, by enhancing AP-1 activity in Jurkat T cells (Figure 4)^37)^. Since IL-2 production in T cells is indicative of immune system activity in vivo, our findings suggest that DIFs could serve as promising leads for developing novel immunosuppressive (and anti-inflammatory) drugs, as well as immunopromotive drugs.

## Antimicrobial activities of DIFs

By the time we discovered the immunoregulatory activities of DIFs, I was convinced that DIFs have numerous biological activities, suggesting their potential as starting points for drug discovery. Meanwhile, I started my position at Juntendo University Graduate School of Health and Sports Science in 2015.

**Figure 5:**
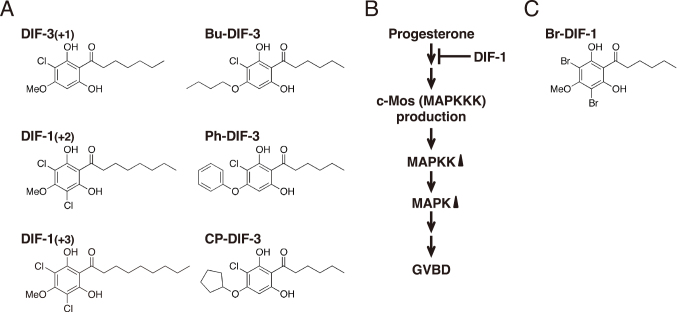
(A) Chemical structures of several DIFs that have antimicrobial activities. DIF-3(+1) and Bu-DIF-3 suppress the infection and growth of *T. cruzi* in vitro with IC_50_ values of 0.5-3 μM^40)^. Ph-DIF-3 and CP-DIF-3 suppress the growth of Gram-positive bacteria, including MRSA and VREs, in vitro with MIC (minimum inhibitory concentration) values of 1-3 μM^43)^. On the other hand, DIF-1(+2) and DIF-1(+3) suppress the growth of *P. falciparum*, including ART-resistant strains, in vitro with IC_50_ values of 0.1-1.5 μM^47, 48)^. (B) Proposed scheme for the anti-meiotic effect of DIF-1 in *Xenopus* oocytes^50)^. Progesterone induces germinal vesicle breakdown (GVBD), at least in part, by stimulating c-Mos (MAPKKK) production and subsequent activation of MAPKK and MAPK. DIF-1 at 20 μM inhibits progesterone-induced GVBD by targeting upstream of c-Mos production^50)^. Abbreviations: MAPK, mitogen-activated protein kinase; MAPKK, MAPK kinase; MAPKKK, MAPKK kinase. (C) Chemical structure of Br-DIF-1. Br-DIF-1 at 0.3-3 μM dose-dependently promotes DMSO-induced cardiomyocyte differentiation in mouse P19CL6 embryonic carcinoma cells^23)^.

*Trypanosoma cruzi* is the protozoan parasite that causes Chagas disease (human American trypanosomiasis)^38, 39)^. To assess the pharmacological potential of DIFs, we examined their anti-*Trypanosoma* activities and found that several DIFs, such as DIF-3(+1) and Bu-DIF-3 (Figure 5A), strongly suppressed the infection and growth of *T. cruzi* in host cells in vitro. Additionally, intraperitoneally administered Bu-DIF-3 suppressed the increase in blood *T. cruzi* concentration in mice^40)^.

On the other hand, due to the increasing number of drug-resistant bacteria, such as methicillin-resistant *Staphylococcus aureus* (MRSA) and vancomycin-resistant *Enterococcus* spp. (VREs), there is a need to search for new antibiotic molecules^41, 42)^. We examined the antibacterial activities of DIFs and found that several DIFs, such as Ph-DIF-3 and CP-DIF-3 (Figure 5A), strongly suppressed the growth of Gram-positive bacteria, including MRSA and VREs^43)^.

Malaria is caused by infection from *Plasmodium* spp., with *P. falciparum* responsible for the deadliest form. The current first-line treatment is artemisinin (ART) combination therapy, which involves administering ART derivatives along with other drugs^44)^. However, due to the global spread of ART-resistant *P. falciparum* strains, there is an urgent need to develop new antimalarial drugs^45, 46)^. We examined the antimalarial activities of DIFs and found that several, such as DIF-1(+2) and DIF-1(+3) (Figure 5A), strongly suppressed the growth of laboratory strains of *P. falciparum*, including ART-resistant strains, in vitro. Additionally, intraperitoneal administration of DIF-1(+2) or DIF-1(+3) suppressed the growth of the rodent malarial parasite *P. berghei* in the blood of mice^47, 48)^.

These findings suggest that DIFs are promising lead compounds for the development of antimicrobial drugs, including treatments for *Trypanosoma* infections, malaria, and bacterial infections.

## Some other biological activities of DIFs

The diversity of biological activities of DIFs is remarkably broad, encompassing the following:

***Anti-meiotic activity***: *Xenopus oocytes* serve as a valuable model for studying meiosis mechanisms. In vitro, oocyte maturation can be induced by progesterone, during which germinal vesicle breakdown (GVBD) (an indicator of meiosis) occurs^49)^. We have demonstrated that DIF-1 can inhibit progesterone-induced GVBD in *Xenopus* oocytes (Figure 5B)^50)^.

***Cardiomyocyte differentiation-promoting activity***: Dimethyl sulfoxide (DMSO) at 1% (v/v) induces cardiomyocyte differentiation in vitro in mouse P19CL6 embryonic carcinoma cells^51)^. We have demonstrated that the activity of DMSO is significantly enhanced in the presence of Br-DIF-1, a derivative of DIF- 1 substituted with bromine instead of chlorine (Figure 5C)^23)^. These findings suggest that Br-DIF- 1 could be beneficial in myocardial regenerative medicine.

## Conclusion

For nearly 40 years, I have focused my research mainly on DIF-1 derived from *D. discoideum* and its derivatives, discovering that DIFs exhibit diverse biological activities. Here, I have provided a brief overview of the biological activities of DIFs and their mechanisms of action. It remains unclear why DIFs display such diverse biological activities, but since these activities can be substantially altered by modifying their side chains, the development of new drugs using DIFs as lead compounds is an attractive research area.

In addition to serving as model organisms for basic biological research, cellular slime molds have garnered attention in recent years as an untapped resource for drug discovery. We have isolated and identified numerous novel compounds derived from cellular slime molds and are currently investigating their biological activities for drug development purposes. For more information on compounds derived from cellular slime molds, including DIFs, please refer to our review^8)^.

## Funding

My work was supported in part by grants from JSPS (KAKENHI), the Japan Science and Technology Agency (JST), the Takeda Science Foundation, the Institute for Fermentation, Osaka (IFO), the Hamaguchi Foundation for Advancement of Biochemistry, the Shimabara Science Promotion Foundation, the Japan Diabetes Foundation, and the Joint Research Program of Juntendo University, Faculty of Health and Sports Science.

## Author contributions

YK contributed to the conception and drafting of the manuscript, along with preparation of the figures.

## Conflicts of interest statement

The author has no conflict of interest to disclose.
